# Yeast in Diabetes

**Published:** 1853-04

**Authors:** 


*Yeast in Diabetes.*—Dr. Wood stated that he had a case of diabetes now under treatment in the Pennsylvania Hospital, which, in the results thus far obtained, was not without interest in a therapeutic point of view. He would first present a brief sketch of the case, drawn up by his young friend, Dr. R. A. F. Penrose, one of the resident physicians of the hospital, and would then offer a few remarks.

"Mary Ann Cain, born in Ireland, a domestic, aged thirty, was admitted into the hospital, November 16, 1852, for palpitation of the heart. Upon examination, the heart was found acting with unusual energy and quickness; but the sounds were normal Her general condition was one of extreme emaciation, her weight eighty-three pounds, the pulse frequent but not strong, the tongue red and smooth. She stated that she suffered much from constant thirst, and had a perpetual desire to eat. Attention was directed to the urine; and it was found that she was passing from 18 to 20 pints daily, of a specific gravity varying from 1036 to 1040. On the addition of yeast it fermented briskly. Boiled, after the addition of solution of sulphate of copper and solution of caustic potash, it yielded a reddish-brown precipitate; boiled with solution of potassa alone, it acquired a dark-brown or bistre tint. The case was clearly one of saccharine diabetes.

"Two days after admission, she was placed upon an animal diet, with non-farinaceous vegetables, and one small biscuit three times a day. Cod-liver oil was also directed, and a *teaspoonful of yeast, three times daily, immediately before meals.*

"22*d.* The quantity of urine now passed in 24 hours was ten pints, and the specific gravity 1022. The thirst and appetite were much diminished. The same treatment continued.

"37*th.* The patient complained of total loss of appetite, and could not take her cod-liver oil. The tongue was extremely red and inclined to dryness, and there was pain on pressure in the epigastrium. The quantity and sp. gr. of the urine were as at last date. The cod-liver oil and animal diet were suspended, and she was placed upon farinaceous drinks with milk. A pill composed of one grain of blue-mass and a quarter of a grain of opium, was directed four times a day, and the yeast was continued.

"30*th.* The patient felt much better, her tongue was moister and less red, and the gastric symptoms were much ameliorated. Not finding milk to agree with her, she lived chiefly on oatmeal gruel, with a soft-boiled egg ocaasionally. The quantity of urine had now been reduced to seven pints in twenty-four hours, and its specific gravity to 1020.

"31*st.* The patient continues as yesterday, the urine having amounted, in the last period of twenty-four hours, to only six pints; the specific gravity not examined."

Much light, Dr. Wood observed, had recently been thrown upon the pathology of diabetes. The disease is now admitted to be characterized by sugar in the blood, the kidneys being only secondarily affected. The experiments of McGregor proved that sugar exists in great excess in the stomach of diabetic patients after eating, and it may readily be supposed to pass thence into the circulation. Bernard has shown that the liver, in its normal action, produces sugar out of the portal blood, and that this sugar passes through the vena cava, right side of the heart, and pulmonary arteries, into the lungs, where, in a healthy state, it is wholly consumed. Excess in the sugar-producing action of the liver, or deficiency in the sugar-consuming action of the lungs, may be followed by the entrance of saccharine matter into the general circulation, and thus give rise to diabetes. The same physiologist proved that, by irritating a certain point of the medulla oblongata, the liver was made to generate a great excess of sugar, which, escaping decomposition in the lungs, entered the arterial circulation, and passed out with the urine.

We thus perceive that there may be various sources of the saccharine impregnation of the blood. *In the first place*, it may arise from some defect in the gastric digestion, in consequence of which farinaceous and other nutritive substances are converted into glucose or grape-sugar, which remains unchanged; or, *secondly*, from hypertrophy or other diseases of the liver causing an over-activity of the sugar-producing function; or, *thirdly*, from diseases of the lungs, impairing their power of consuming the sugar; or, *fourthly*, from irritation of the nervous centre in the medulla oblongata which appears to control the action of the liver in relation to this product; or, *lastly*, from two or more of these sources combined.

Now, in the case before us, no organic affection of the liver or of the lungs could be detected, and there was no reason to suspect the medulla oblongata; but the smooth, reddened state of the tongue, and the epigastric tenderness, seemed to point directly to the stomach as the seat of the disease.

In the number of the *Edinburg Monthly Journal of Medical Science*, for October last, it it is stated that Dr. Gray, of Glasgow, had been induced to make trial of *rennet* in a case of diabetes, in the hope that, as this body converts sugar out of the body into lactic acid, it might be found to produce a similar change within the stomach, and the lactic acid thus generated might be eliminated from the system, or rather decomposed by the respiratory process. A teaspoonful of rennet was given three times a day. In eight days, the specific gravity of the urine was reduced to 1025, with but a trace of sugar; in twenty-five days, the quantity was four pints, and the density 1022.5, and no sugar could be detected. At the end of six weeks, the urine remained free from sugar, and the patient had so far improved in health and strength as to return to his work.

It occurred to me, observed Dr. Wood, from the results of this case, that yeast might prove equally beneficial, by causing a decomposition of the sugar in the stomach such as it is well known to occasion out of the body, resulting in the production of acetic acid. Being under the impression that in the case now reported to the College, the primary disease probably resided in the stomach, and that the diabetic sugar was generated there, I determined to try the effects of this remedy. The patient had been two days in the house before she was placed under treatment, and during this period no change had taken place in the quantity or character of the urine. It will have been noticed that quickly after the commencement of treatment, a very great change took place in both these respects; the quantity of urine being reduced, in the course of four days, from twenty pints to ten daily, and the specific gravity from 1036 or 1040 to 1022. But the almost exclusive use of animal food may be supposed to have contributed to this result. In consequence of the gastric inflammation, it was necessary to suspend this diet, and to allow the patient to use farinaceous food, the yeast being continued. So far from any increase of urine in consequence of this change of diet, its quantity was still further reduced, so that, upon the last day upon which it was examined, it did not exceed six pints, while the specific gravity was as low as 1020, the quantity having been reduced from twenty pints to about double that of health, and the density from 1040 to that of normal urine. There can be no doubt whatever that the sugar has been very greatly diminished; and there is no cause apparent to which the result can be ascribed except the use of yeast.

What may be the further progress of the case, cannot, of course, be foreseen. Even should we succeed in preventing altogether the elimination of sugar with the urine, it does not follow that the case will end in recovery. The remedy is addressed only to one of the effects; a very important effect, it must be admitted, and itself capable of producing great mischief, but still by no means the whole disease. Nevertheless, if, by the steady use of a remedy so little disagreeable as yeast, we can prevent the abnormal production of sugar, and the exhausting effects on the system of the excessive secretion of urine occasioned by it, we shall have gained one great point. We shall at least gain time for accurately investigating the source of the evil, and applying such remedies as may offer a reasonable hope of permanent benefit.

Dr. Wood observed, finally, that he should probably take occasion, at a future meeting of the College, to report the further progress of the case; and should have been in less haste at present, had he not thought that the remedial measure had some claims to notice, and been desirous that it should, as quickly as possible, receive an ample trial, so that its merits might be conclusively tested.—*Trans. College of Physicians, Philadelphia.*

